# Effects of hypochlorous acid mouthwash on salivary bacteria including *Staphylococcus aureus* in patients with periodontal disease: a randomized controlled trial

**DOI:** 10.1186/s12903-023-03358-4

**Published:** 2023-09-28

**Authors:** Ying-Chu Lin, Cheng-Feng Tsai, Hsiao-Ling Huang

**Affiliations:** 1 School of Dentistry, College of Dental Medicine, Kaohsiung Medical University, No.100, Shih-Chuan 1st Road, Kaohsiung, 80708 Taiwan; 2 Wenhsin Dental Clinic, 1F, No. 182, Zhongzheng 2nd Rd., Xinxing Dist, Kaohsiung, Taiwan; 3 Department of Oral Hygiene, College of Dental Medicine, Kaohsiung Medical University, No.100, Shih-Chuan 1st Road, Kaohsiung, 80708 Taiwan

**Keywords:** Antibacterial ability, Total bacterial count, Hypochlorous acid, Mouthwash, Intervention, Bacterial growth estimation

## Abstract

**Background:**

The effects of a low concentration of hypochlorous acid (HOCl) mouthwash on salivary bacteria remained unclear. We aimed to evaluate the antibacterial effects of 100 ppm HOCl mouthwash on salivary bacteria, including *Staphylococcus aureus* (*S. aureus*), in patients with periodontal disease (PD).

**Methods:**

Patients with PD were randomized into mouthwash-only (MW, *n* = 26) and mouthwash with periodontal flosser (MWPF, *n* = 27) groups. Patients without PD were selected for the control group (*n* = 30). *S. aureus* culture and saliva samples (before and after the intervention) were collected for bacterial DNA extraction. A real-time polymerase chain reaction assay and serial dilutions of *S. aureus* culture and saliva samples were used to measure the salivary bacteria total count (SBTC) and confirm the antibacterial effects of the mouthwash using *S. aureus.*

**Results:**

No significant difference in demographic data was observed among the three groups. Before the intervention, the baseline SBTC of the MW and MWPF groups was significantly higher than that of the control group. After the mouthwash rinses, the SBTC data significantly changed in the MW and MWPF groups only (by 62.4% and 77.4%, respectively). After the base-2 log-transformation of the SBTC data, a similar trend was observed. Linear regression revealed that baseline SBTC and the MWPF intervention significantly affected SBTC reduction percentage by volume. After incubation with 10% (v/v) of mouthwash, the survival rates of 10^6^ and 10^7^ colony-forming units/mL of *S. aureus* were 0.51% ± 0.06% and 1.42% ± 0.37%, respectively.

**Conclusions:**

These study results indicated that 100 ppm HOCl mouthwash treatment could effectively reduce SBTC in patients with PD and the abundance of *S. aureus*. It provides that the HOCl mouthwash can be an option for individuals to help control SBTC, especially in patients with PD.

**Trial registration:**

The study protocol was approved by the Institutional Review Board of Kaohsiung Medical University Hospital (KMUHIRB-F(I)-20200042) on 20/03/2020 and retrospectively registered at *ClinicalTrial.gov* (NCT05372835) on 13/05/2022.

## Introduction

Periodontal disease (PD) is a common oral disease in adults (individuals aged 30 years or older). The symptoms of PD are inflammation of the gingiva and periodontium [1]. Poor oral hygiene is a risk factor for PD [2]. The accumulation of dental plaque—especially when pathogenic bacteria are dominant—can lead to progressive periodontitis and the loss of teeth [3, 4]. Controlling the bacteria in the mouth is key to maintaining oral health [5]. The use of mouthwash and periodontal flossers can inhibit oral bacterial growth and increase the concentration of beneficial bacteria, thereby promoting dental health and preventing gum problems [6–8]. Mouthwash can effectively reduce oral bacterial counts in patients with periodontitis; however, the effects and efficacy of different mouthwash components on periodontitis remain unclear, and few studies have demonstrated the efficacy of such products [9]. Mouthwashes that contain ingredients with antibacterial properties, particularly those that can effectively reduce the number of microorganisms related to PD, have become popular.

Two key concerns regarding the use of mouthwash are its unpleasant taste and its potential to cause tooth staining [1]. Mouthwashes containing chemicals such as chlorhexidine and hypochlorous acid (HOCl) have been used to reduce the accumulation of oral bacteria and pathogens [1, 10, 11]. HOCl is a nonantibiotic antibacterial solution [12]. A stabilized HOCl solution was observed to effectively and rapidly kill most microorganisms it came in contact with in vitro [13, 14]. HOCl has potent anti-inflammatory and cytoprotective effects [13–15]. HOCl removes the outer polymer matrix of biofilms formed by microorganisms in a manner akin to physically scraping them off [13]. HOCl consists of a group of reactive oxygen species [16]. It exerts broad antibacterial effects on multiple Gram-positive and Gram-negative bacteria and has few side effects. HOCl does not irritate mucous membranes or result in pigmentation on the surface of teeth or restorations [16]. These studies support that HOCl mouthwash can be effective disinfection in dental care and oral hygiene.

Saliva serves as a reservoir of microorganisms and plays a key role in regulating bacterial colonization in the various oral structures [17]. Mouthwash is already known to contribute to the decrease of oral bacteria numbers and dental plaque accumulation. Previous in vitro studies indicate that HOCl mouthwash at a concentration of 250—500 ppm or lower concentration is an effective antimicrobial agent to reduces the viability of pathogenic bacteria of oral diseases without having the significant side effects [11, 18–20]. However, rare in *vivo* studies report the antibacterial effects of HOCl mouthwash on salivary bacteria in patients with PD, especially in lower concertation of HOCl. Recently, a new mouthwash called Chlogen has recently become available in Taiwan. Chlogen contains HOCl at a concentration of 100 ppm, which is relatively low. Whether Chlogen is effective at controlling salivary bacteria is unclear. A randomized controlled trial was conducted to investigate the antiseptic effects of Chlogen on salivary bacterial total count (SBTC) in patients with PD with and without concurrent use of periodontal flosser. We also evaluated whether the intervention’s effects differed significantly among these three groups. A commercialized bacterial strain (*Staphylococcus aureus*) with pure culture was used to compare the antiseptic effects of Chlogen.

## Materials and methods

### Ethics statement

The study protocol was approved by the Institutional Review Board of Kaohsiung Medical University Hospital (KMUHIRB-F(I)-20200042) and retrospectively registered at *ClinicalTrial.gov* (NCT05372835). The study protocol followed the Declaration of Helsinki. The participation was voluntary and anonymous after the informed consent obtained.

### Participants

**Fig. 1:**
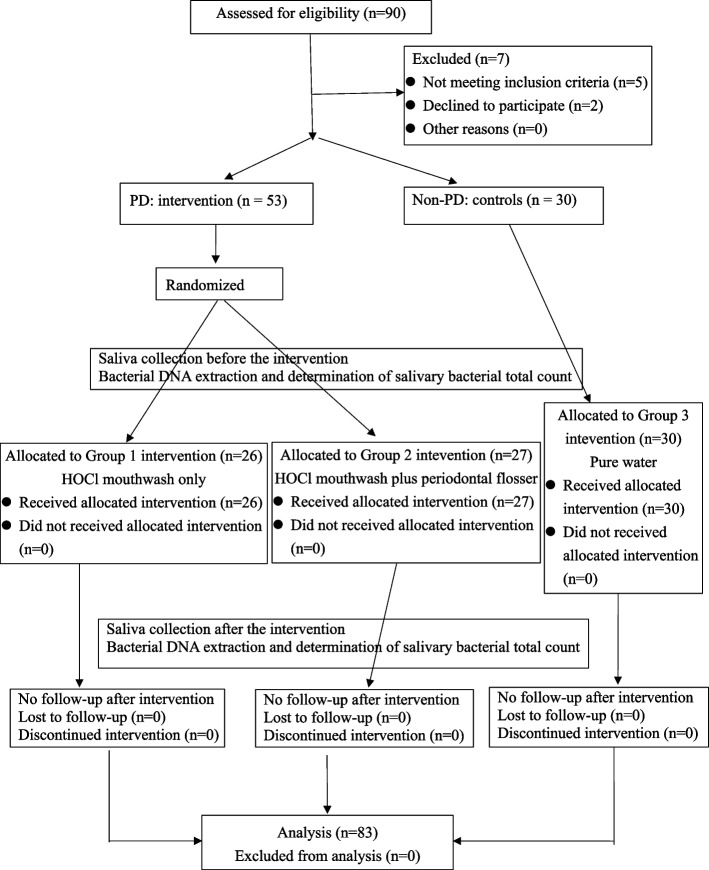
The CONSORT flowchart

This was a double-blind (participants and lab investigation), randomized, parallel clinical trial enrolled outpatients at a private dental clinic (Fig. 1). Participants were aged between 35 and 70 years and provided written informed consent. Information on health histories and use of dental cleaning tools, such as mouthwash, was collected. Full-mouth dental examination records were checked by the same dentist. Gingivitis was defined as > 10% of tooth sites bleeding upon probing (BOP) and/or gingival bleeding (GI) and without probing pocket depth (PPD) or clinical attachment loss (CAL) > 3 mm. Periodontitis was defined as > 10% of teeth or > 15% of tooth sites with BOP and PPD or CAL ≥ 5 mm [21, 22]. The PDs of intervention were gingivitis and periodontitis. Patients without PD were enrolled into a control group. Patients who did not wish to participate, who had used antibiotics or immunosuppressive drugs within the last 6 months, who were pregnant, who smoked regularly, or who had a history of cancer or systemic disease were not included. Sample size was calculated using the mean difference in SBTC with base 2-log transformed data (effect size (ES) = 0.8, α = 0.05, and power = 0.8). Estimated sample size was 19 for each group. The IRB project was ended on schedule. In total, 53 patients with PD were randomly divided into two groups; one group (*n* = 26) used mouthwash only (Taiwan Patent No. M616466), and the other group (*n* = 27) used a mouthwash and periodontal flosser (Taiwan Patent No. M590033). A total of 30 individuals without PD were enrolled into the control group and gargled using 15 mL of pure water.

### Data collection

Before saliva collection, participants rinsed with 5 mL of water. Before and after the intervention, participants were asked to expectorate for a maximum of 3 min into a 50-mL sterile centrifugation tube (Creative Biotechnology, Taipei, Taiwan). The collected specimens were immediately placed in a portable ice box for storage and sent to a laboratory for same-day bacterial DNA extraction. The volume and weight of each saliva sample were recorded.

### SBTC analysis

*S. aureus* ATCC 29213 was used as a reference strain in all experiments. The protocol for salivary bacterial DNA extraction, RT-PCR reaction, and bacterial growth curve estimation is described in our previous study [23] and *ClinicalTrial.gov* database ID: NCT05372835. After bacterial DNA extraction, real-time polymerase chain reaction (RT-PCR) was used to quantify the SBTC data (SBTC/mL and SBTC/g) in the samples. The RT-PCR assay of each sample was conducted in triplicate to calculate the SBTC data of each sample by using the equation created in our previous study [23]. Human blood DNA was used as a negative control group. The coefficient of variation of the threshold was between 2 and 11%.

### Antibacterial activity of the mouthwash

After serial dilution, *S. aureus* solutions [0, 10^1^, 10^2^, 10^3^, 10^4^, 10^5^, and 10^6^ colony-forming units (CFU)/mL] were centrifuged at 2000 rpm for 5 min to concentrate the bacteria. The liquid was removed, 180 µL of fresh culture medium was added to resuspend the bacteria, and the cell suspension was aliquoted in 96-well plate. Next, 20 µL of the mouthwash was added to each well. The mixture was incubated at 37 °C for 2 h. Finally, 10 µL of CCK-8 reagent (CCK-8, Omics Bio, New Taipei City, Taiwan) was added and the mixture was agitated. The 96-well plate was incubated at 37 °C for 2 h, and the absorbance optical density (OD) value at 450 nm was measured using an ELISA reader. Each experiment was performed in triplicate. A standard growth curve was established for the measurement of serial dilution of the bacteria in the mixture and the bacterial survival rate.

### Statistical analysis

After the data collection and checking were complete, the finalized debugged files were linked as a full dataset for statistical analysis with JMP statistical software (version 11, SAS Institute Inc. Cary, North Carolina, U.S.). Data were presented in frequency distribution tables and as percentages, means, and standard deviations. Considering the distribution of SBTC data, nonparametric statistical methods and base 2–log-transformed data were used to identify differences in the numerical data. Linear regression analysis was conducted to assess the factors contributing to percentage changes in SBTC after the intervention.

## Results

A total of 83 participants was recruited into the present study. The mean age of participants was 58.0 ± 13.1 years. No significant differences in age (*p* = 0.21), man-to-woman ratio (*p* = 0.056), and collected saliva sample weight (*p* = 0.17) and volume (*p* = 0.53) were observed among the MW, MWPF, and control groups (Table 1). The average baseline bacterial counts by saliva volume in the MW, MWPF, and control groups were 2.97 ± 8.73 × 10^8^, 7.99 ± 4.82 × 10^8^, and 2.21 ± 4.26 × 10^6^ CFU/mL, respectively, and the corresponding bacterial counts by saliva weight were 3.40 ± 1.06 × 10^9^, 8.41 ± 3.90 × 10^9^, and 2.29 ± 4.25 × 10^7^ CFU/g, respectively. The baseline SBTC data in the MWPF and MW groups were significantly higher than that in the control group (*p* < 0.001). However, no significant difference between the MWPF and MW groups was observed. The SBTC data normalized through a log transformation by using base 2, and similar statistical results were obtained.

**Table 1:** Baseline information of the mouthwash-only group, mouthwash and periodontal flosser group, and control group

Variables	Mouthwash (*n* = 26)	Mouthwash and flosser (*n* = 27)	Control (*n* = 30)	p*
	n %	n %	n %	
Age (years)^a^	61.0 ± 10.8	55.6 ± 11.9	57.4 ± 16.0	0.211
Gender				
Male	17 65.4	12 44.4	10 33.3	0.056
Female	9 34.6	15 55.6	20 66.7	
Saliva weight (g)^a^	1.0 ± 0.3	1.2 ± 0.7	1.0 ± 0.5	0.165
Saliva volume (mL)^a^	1.1 ± 0.3	1.2 ± 0.7	1.0 ± 0.5	0.534
Bacterial count (CFU/mL)^a^	2.97 ± 8.73 × 10^8^	7.99 ± 4.82 × 10^8^	2.21 ± 4.26 × 10^6^	< 0.001
Bacterial count (CFU/g)^a^	3.40 ± 1.06 × 10^9^	8.41 ± 3.90 × 10^9^	2.29 ± 4.25 × 10^7^	< 0.001
Log-transformed data with a base of 2				
Bacteria count (CFU/mL)^a^	17.2 ± 2.1	17.2 ± 2.3	14.7 ± 2.2	< 0.001
Bacteria count (CFU/g)^a^	17.3 ± 2.1	17.2 ± 2.3	14.8 ± 2.2	< 0.001

Mouthwash and flosser: mouthwash in combination with a periodontal flosser

^a^Data are expressed as means ± standard deviation

^*^ *p* value calculated using Fisher’s exact test for frequency distributions and the Kruskal–Wallis test for numeric data

SBTC before and after the intervention is presented in Table 2. SBTC decreased significantly in the MW and MWPF groups by nonparametric statistics but did not reduce significantly in the control group. The changes in SBTC in the MW and MWPF groups were also significantly larger than those in the control group (*p* < 0.001), whose SBTC increased. The % decrease in SBTC by volume and by weight was significantly lower after the intervention in the MWPF group (− 77.4% ± 25.7% and − 74.8% ± 32.3%, respectively) (*p* < 0.001) and MW group (− 62.4% ± 31.9% and − 61.6% ± 33.9%, respectively) (*p* < 0.001) but higher after the intervention in the control group (259.5% ± 1105.5% and 289.9% ± 137.0%, respectively).

**Table 2:** Changes in bacterial counts by saliva weight (CFU/g) and saliva volume (CFU/mL) in the mouthwash-only group, mouthwash and periodontal flosser group, and control group

Variables	Mouthwash	Mouthwash and flosser	Control	
	Mean ± SD (*n* = 26)	Mean ± SD (*n* = 27)	Mean ± SD (*n* = 30)	*p* ^b^
Bacterial Count (CFU/mL)				
Pretest	2.97 ± 8.73 × 10^8^	7.99 ± 34.8 × 10^8^	2.21 ± 4.26 × 10^6^	
Posttest	3.12 ± 15.13 × 10^7^	1.43 ± 3.06 × 10^7^	3.82 ± 16.3 × 10^8^	
Change	− 2.88 ± 8.98 × 10^8^	− 8.48 ± 36.1 × 10^8^	3.03 ± 16.1 × 10^8^	< 0.001
*p* ^*a*^	< 0.001	< 0.001	0.337	
Change %	− 62.4 ± 31.9	− 77.4 ± 25.7	259.5 ± 1105.5	< 0.001
Bacterial Count (CFU/g)				
Pretest	3.40 ± 1.06 × 10^9^	8.41 ± 3.90 × 10^9^	2.29 ± 4.25 × 10^7^	
Posttest	2.57 ± 4.58 × 10^6^	1.69 ± 4.40 × 10^6^	2.97 ± 12.7 × 10^7^	
Change	− 3.64 ± 11.0 × 10^8^	− 9.07 ± 4.06 × 10^9^	0.59 ± 110 × 10^6^	< 0.001
*p* ^*a*^	< 0.001	< 0.001	0.001	
Change %	− 61.6 ± 33.9	− 74.8 ± 32.3	289.9 ± 137.0	< 0.001

*SD* Standard deviation; change % = 100% × (posttest − pretest)/pretest

^*a*^ *p* value calculated using the Wilcoxon rank-sum test

^b^*p* value calculated using the Kruskal–Wallis test and Tukey’s test

Log-transformed SBTC after the intervention is presented in Table 3. The statistical analysis with log-transformed data were similar to the original data (*p* < 0.001). The % decrease in SBTCs by volume and by weight were significantly lower after the intervention in the MW group (− 9.7% ± 8.9% and − 9.6% ± 8.9%, respectively) (*p* < 0.001) and MWPF group (− 13.4% ± 9.6% and − 13.35% ± 10.1%, respectively) (*p* < 0.001) but not in the control group.

**Table 3:** Log-transformed changes in bacterial counts by saliva weight (CFU/g) and saliva volume (CFU/mL) in the mouthwash group, mouthwash and periodontal flosser group, and control group

Variables	Mouthwash	Mouthwash and flosser	Control	
	Mean ± SD (*n* = 26)	Mean ± SD (*n* = 27)	Mean ± SD (*n* = 30)	*P* ^b^
Bacterial Count (CFU/mL)*				
Pretest	17.2 ± 2.1	17.2 ± 2.3	14.7 ± 2.2	
Posttest	15.5 ± 2.2	15.1 ± 1.9	14.7 ± 2.7	
Change^b^	− 1.7 ± 1.7	− 2.4 ± 2.0	− 0.4 ± 2.0	< 0.001
*p* ^a^	< 0.001	< 0.001	0.421	
Change %	− 9.7 ± 8.9	− 13.4 ± 9.6	− 1.9 ± 12.1	< 0.001
Bacterial Count (CFU/g)*				
Pretest	17.3 ± 2.1	17.2 ± 2.3	14.8 ± 2.2	
Posttest	15.5 ± 2.2	15.1 ± 1.8	14.6 ± 2.7	
Change^b^	− 1.7 ± 1.8	− 2.4 ± 2.1	− 0.5 ± 2.1	< 0.001
*p* ^a^	< 0.001	< 0.001	0.199	
Change %	− 9.6 ± 8.9	− 13.3 ± 10.1	− 2.6 ± 12.5	< 0.001

*SD* standard deviation; change % = 100% × (posttest − pretest)/pretest

^*^Log-transformed with a base of 2

^a^ *p* value calculated using the Wilcoxon rank-sum test

^b^ *p* value calculated using the Kruskal–Wallis test and Tukey’s test

Multivariate linear regression analysis of log-transformed SBTC data (Table 4) revealed that baseline SBTC (β =  − 0.02, 95% confidence interval [CI] =  − 0.03 to − 0.005 and β =  − 0.06, 95% CI =  − 0.12 to 0.00, respectively) was significantly correlated with % decreases in SBTC. The MWPF (vs. control: β =  − 0.07, 95% CI =  − 0.13 to − 0.10) group significantly affected the % decrease in SBTC by volume, but it was a borderline significance to affect the % decrease in SBTC by weight. Other factors, namely MW group (vs. controls: β =  − 0.03, 95% CI =  − 0.09 to 0.03 and β =  − 0.02, 95% CI =  − 0.08 to 0.05, respectively), age (β =  − 0.0002, 95% CI =  − 0.002 to 0.001 and β =  − 0.0001, 95% CI =  − 0.002 to 0.002, respectively) and sex (β = 0.04, 95% CI =  − 0.01 to 0.08 and β = 0.04, 95% CI =  − 0.01 to 0.09, respectively) did not significantly affect the % decrease in SBTC data.

**Table 4:** Linear regression–estimated intergroup differences in base-2 log-transformed percent changes in bacterial counts by saliva weight (CFU/g) and saliva volume (CFU/mL)

Variables	β	(95% CI)	Effect size^b^	*p*
Bacterial Count (CFU/mL)*				
Mouthwash group^a^	− 0.03	(− 0.09, 0.03)	0.73	0.348
Mouthwash and flosser group^a^	− 0.07	(− 0.13, − 0.01)	1.05	0.022
Baseline SBTC	− 0.02	(− 0.03, − 0.005)		0.005
Age	− 0.0002	(− 0.002, 0.001)		0.736
Gender	0.04	(− 0.01, 0.08)		0.123
Bacterial Count (CFU/g)*				
Mouthwash group^a^	− 0.02	(− 0.08, 0.05)	0.64	0.595
Mouthwash and flosser group^a^	− 0.06	(− 0.12, 0.00)	0.94	0.064
Baseline SBTC	− 0.02	(− 0.03, − 0.01)		0.002
Age	− 0.0001	(− 0.002, 0.002)		0.907
Gender	0.04	(− 0.01, 0.09)		0.095

*SBTC* salivary bacterial total count

^*^Log-transformed with a base of 2

^a^Reference group: control group

^b^Effect size calculated using mean difference of change between baseline and post intervention

A standard curve was established through a CCK-8 assay, and *S. aureus* growth was described as bacterial count log value = 3.515 + 0.556 × OD value. The survival rates of 10^6^ and 10^7^ CFU/mL of *S. aureus* were 0.51% ± 0.06% and 1.42% ± 0.37%, respectively.

## Discussion

Present study found the SBTC of patients with PD in the MW and MWPF groups was significantly higher than that of the control group before the intervention. After the HOCl mouthwash intervention 5 min, the SBTC data significantly decreased in the MW and MWPF groups. After base-2 log-transformation of the SBTC data, a similar trend was observed. Multivariate linear regression revealed that baseline SBTC and the MWPF intervention significantly affected SBTC reduction percentage by volume. Previous study report that *S. aureus* is a common pathogenic bacteria found in the oral cavity of patients with PD [24]. Present study showed that 10% (v/v) mouthwash killed more than 98% of *S. aureus* by an in vitro assay. Although *S. aureus* used in this study was not identified from the study participants, these study results could partially support that 100 ppm HOCl mouthwash could effectively reduce the salivary bacterial load and *S. aureus* in patients with PD. However, previous study show that antimicrobial susceptibility of the same disinfection agent to planktonic and sessile cells are different [25]. Further studies are needed to test the antimicrobial effects of HOCl mouthwash on dental biofilm.

Previous studies show that SBTC by volume can be as high as 10^8^ or 10^9^ CFU/mL [26, 27]. SBTC by volume in patients with PD was significantly higher than in individuals with adequate oral health [28]. SBTC by volume decreased by 33% and 58% after participants gargled for 30 s with 500 ppm HOCl and 0.2% chlorohexidine, respectively [29]. In the present study, gargling with 100 ppm HOCl mouthwash for 5 min decreased SBTC by volume by 62.4% and 77.4% in the MW and MWPF groups, respectively. Before the intervention, SBTC by volume was significantly higher in patients with PD than in the control group. In the intervention groups, SBTC by volume was as high as 10^8^ CFU/mL and by weight was as high as 10^9^ CFU/g. Our study results are consistent with those of previous studies [26, 27]. The difference in percent decrease in SBTC by volume can be partially explained by the duration of use and concentration of HOCl.

Multivariate linear regression analysis revealed that the effect sizes of the MW (ES = 0.73) and MWPF (ES =1.05) groups were medium and large by the Cohen classification [30], respectively. In the present study, SBTC by volume in the MW and MWPF groups decreased by 62.4% and 77.4%, respectively, indicating that the antibacterial effect of the mouthwash plus periodontal flosser (MWPF group) higher than that of the mouthwash only (MW group). The decrease in SBTC by volume was 15% larger in the MWPF group than in the MW group but nonsignificant. For the base 2-log-transformed SBTC data, linear regression analysis revealed that the MWPF group (vs. controls) significantly affected the percentage decrease in SBTC by volume. Clinical studies have demonstrated that periodontal flossers facilitate greater contact between mouthwash and oral bacteria, thereby strengthening the antibacterial effects of mouthwash. The use of both a dental flosser and a manual toothbrush is significantly more effective for improving gingival health than a manual toothbrush alone [31]. These findings in combination with our results suggest that a periodontal flosser enhances the antibacterial effects of mouthwash.

PD is caused by an increase in dental plaque and inflammation. Early-stage PD, involving gingivitis, is reversible [32]. When dental plaque accumulates, gingivitis progresses to periodontitis, which is the irreversible destruction of periodontal tissue and alveolar bone [3]. Elevated gingival crevicular fluid miRNA expression is a potential biomarker for periodontitis or periodontal inflammation area [33]. In the present study, HOCl mouthwash effectively and rapidly decreased SBTC. It is possible that the HOCl mouthwash can contribute to the prevention and management of PD. Biomarkers can be used to detect PD at an early stage, thereby weakening the effects of PD on quality of life. These study results suggest that PD can be identified at an early stage by biomarkers and further to be prevented through the effective removal of plaque biofilm by using the mouthwash. Well-designed studies with large samples are required to support our hypotheses.

In one study, HOCl mouthwash had no direct antibacterial effect at 7 h after use, and bacterial count returned to baseline within 1 h of use [29]. Because of the limited observation time, we were unable to determine the duration of the bactericidal or bacteriostatic effects of the mouthwash in the oral cavity. More studies are required to investigate how the mouthwash in the present study can most effectively be used.

During the oral bacterial DNA extraction, some saliva samples were contaminated with small pieces of food residue. The food residue may have increased the variability of the antibacterial effects of mouthwash on SBTC by weight. This may partially explain why the MWPF intervention was not a significant factor in the regression analysis for the reduction in SBTC by weight.

A broad variety of mouthwash is available in the market. The potential unfavorable side-effects of mouthwash are cell cytotoxicity, bacterial resistance, tooth staining, and unpleasant taste [10, 34]. Some of these side-effects can be correlated with the CHX mouthwash [34]. Compared with the usage frequency of mouthwash with CHX, it is not common use of HOCl mouthwash [6]. Previous study indicate that HOCl can be produced from human immune cells and it has no such side-effects on the structure of oral cavity [35]. Present study showed that patients with PD could significantly reduce the SBTC data after the usage of HOCl mouthwash in a short time period. These study results suggest that 100 ppm HOCl mouthwash can be a good alternative option for patients with PD helping to reduce the SBTC.

This study has several limitations. First, data on the effects of durations of use and concentrations of HOCl mouthwash were not collected. Therefore, the most effective method of using the present mouthwash remains unclear. Although 100-ppm HOCl mouthwash is known to be nontoxic to cells in our previous study (data not shown), the antibacterial efficacy of the mouthwash on individual periodontitis-causing bacteria was not investigated in this study. Numerous types of bacteria are present in saliva, and the environment in which bacteria grow can vary depending on bacterial strain; further research is required to determine the antibacterial effects of mouthwash on salivary specific periodontal pathogens.

## Conclusions

The baseline SBTC was significantly higher in patients with PD and contributed to the percentage reduction of SBTC data by multivariate regression analysis. The treatment of 100 ppm HOCl mouthwash could effectively decrease the SBTC in patients with PD and the level of *S. aureus* by an in vitro study, respectively. We suggest that HOCl mouthwash is an option for individuals to help control bacteria in the mouth, especially in patients with PD.

## Data Availability

Data that support the findings of this study are available from the corresponding author upon reasonable request.

## Abbreviations

CFU: Colony-forming unit
ES: Effect size
HOCl: Hypochlorous acid
MW: Mouthwash
MWPF: Mouthwash and periodontal flosser
OD: Optical density
PD: Periodontal disease
RT-PCR: Real-time polymerase chain reaction
S. aureus: Staphylococcus aureus
SBTC: Salivary bacterial total count
